# Metamagnetic transition in single-crystalline UIr$_{2}$Si$_{2}$

**DOI:** 10.1038/s41598-023-41723-z

**Published:** 2023-09-07

**Authors:** Maria Szlawska, Magdalena Majewicz, Dorota A. Kowalska, Dariusz Kaczorowski

**Affiliations:** Institute of Low Temperature and Structure Research, Polish Academy of Sciences, 90-950 Wrocław, Poland

**Keywords:** Magnetic properties and materials, Phase transitions and critical phenomena

## Abstract

A single crystal of the ternary uranium silicide UIr$_{2}$Si$_{2}$ was studied by means of of X-ray diffraction, magnetization, heat capacity and electrical transport measurements. The studied compound orders antiferromagnetically at the Néel temperature of 5.5 K and undergoes a metamagnetic transition at 1.8 K in a field of 1.52 T. The metamagnetic transition has a spin-flop character.

## Introduction

The wide variety of physical behaviors observed in U-based intermetallic compounds results mostly from the interactions of electrons from the conduction band with those from the partially delocalized 5*f* electron shell. Among them, the $\hbox {UT}_{2} \hbox {M}_{2}$ family (T stands for *d*-electron transition metal, while M is Si or Ge atom) has attracted particular attention. The compounds belonging to this group can crystallize with two closely related types of the unit cell, namely the body-centered $\hbox {ThCr}_{2} \hbox {Si}_{2}$ (space group *I*4/*mmm*) or the primitive $\hbox {CaBe}_{2} \hbox {Ge}_{2}$ type (*P*4/*nmm*). Both structures are derivatives of the $\hbox {BaAl}_{4}$ type, in which U atoms occupy the Ba site, while T and M atoms are located on the Al site. They can be described as a sequence of planes composed of the same types of atoms stacked along the tetragonal *c* axis. The $\hbox {ThCr}_{2} \hbox {Si}_{2}$ type unit cell is described as U–M–T–M–U–M–T–M–U, while the $\hbox {CaBe}_{2} \hbox {Ge}_{2}$-type is described as U–M–T–M–U–T–M–T–U^1^. In the latter type, which is centrosymmetric, the U atoms are located in a non-centrosymmetric position. This makes the study of the compounds that adapt such kind of the crystal structure particularly interesting due to the phenomena that can arise from the splitting of the Fermi surfaces by spin-orbit coupling. On the other hand, it should be noted that since $\hbox {UT}_{2} \hbox {M}_{2}$ compounds can adapt two closely related types of crystal structures, they are prone to the appearance of crystallographic disorder at the T and M positions, which can strongly influence their physical properties^2,3^.

The Si-bearing members of the $\hbox {UT}_{2} \hbox {M}_{2}$ family exhibit a wide variety of physical properties. The most studied member of the family, $\hbox {URu}_{2} \hbox {Si}_{2}$ undergoes a transition at low temperatures to the so-called “hidden-order” state and further to the superconducting state^4^. The compounds with T = Fe, Os, Re are Pauli paramagnets^5,6^, while those with T = Cr, Mn, Co, Ni, Cu, Rh, Pd, Ir, Pt, Au show long-range magnetic ordering^7–16^. It is noteworthy that in all of the above-mentioned phases, the magnetic moments are carried only on U atoms (except for the Mn-bearing one). Due to the fact that their unit cell is strongly elongated along the *c* axis, and the magnetocrystalline anisotropy is strong in these compounds, the magnetic moments are oriented parallel to the *c* axis. Most members of the $\hbox {UT}_{2} \hbox {Si}_{2}$ group crystallize with the $\hbox {ThCr}_{2} \hbox {Si}_{2}$-type unit cell. The exceptions are $\hbox {UPt}_{2} \hbox {Si}_{2}$ and $\hbox {UIr}_{2} \hbox {Si}_{2}$, which possess the $\hbox {CaBe}_{2} \hbox {Ge}_{2}$ type. Inealastic X-ray scattering experiments have shown that the *f* electrons in compounds of the former type have a dual nature. Their electronic ground state is basically $\Gamma ^{(1)}_{1}$ + $\Gamma _{2}$ quasi-doublet that stems from the $5f^{2}$ configuration, however an admixture of the $5f^{3}$ configuration brings about some itinerancy of the 5*f* electrons that governs the differences in the magnetic behaviors of the $\hbox {UT}_{2} \hbox {Si}_{2}$ compounds^17^.

$\hbox {UIr}_{2} \hbox {Si}_{2}$ was described in the first literature report as an antiferromagnet with the Néel temperature $T_{N} = 4.9$ K, that exhibits a metamagnetic transition at low temperatures in a magnetic field $\mu _0 H_c$ = 2.2 T applied along the *c* axis^14^. In the later article, Vernière et al.^18^ slightly different values of the Néel temperature (6 K) and the metamagnetic transition field (1.5 T) were reported. The differences were attributed to a slight deviation of the composition of the investigated crystal from the nominal one (presence of Si atoms on Ir positions), which was expected to influence the exchange interactions between magnetic U ions^18^. Subsequent neutron diffraction experiments on $\hbox {UIr}_{2} \hbox {Si}_{2}$ revealed that the magnetic moments, have a small magnitude of $0.1 \ \mu _{B}$ and are oriented along the *c* axis. They form ferromagnetically ordered planes, coupled antiferromagnetically^19^. The other important feature of $\hbox {UIr}_2 \hbox {Si}_2$ is the strong influence of the crystal field on its properties^14,20^. The experiments performed under pressure conditions showed that the $T_{N}$ does not change up to 6 GPa^21^. More recent ultrasonic measurements brought to light the existence of electric quadrupolar contributions in $\hbox {UIr}_{2} \hbox {Si}_{2}$^22^.

Until now, there has been no comprehensive study of the magnetic phase transitions in $\hbox {UIr}_2 \hbox {Si}_2$. This study focuses on the magnetization, electrical resistivity, and heat capacity measurements conducted on high-quality single-crystalline specimens in the vicinity of the metamagnetic phase transition. We present the magnetic phase diagram developed from the results of our studies.

## Results

### Crystal structure and composition

Energy-dispersive X-ray spectroscopy (EDX) measurements indicated that the prepared crystal was homogeneous and single phase. The EDX analysis yielded the composition to be U—18.7(6) at. %, Ir—37.9(5) at. %, Si—43.3(5) at. %, which corresponds to the formula $\hbox {U}_{0.9\pm 0.1} \hbox {Ir}_{1.9\pm 0.1} \hbox {Si}_{2.2 \pm 0.1}$ close to the nominal one, with an excess of Si.

**Figure 1:**
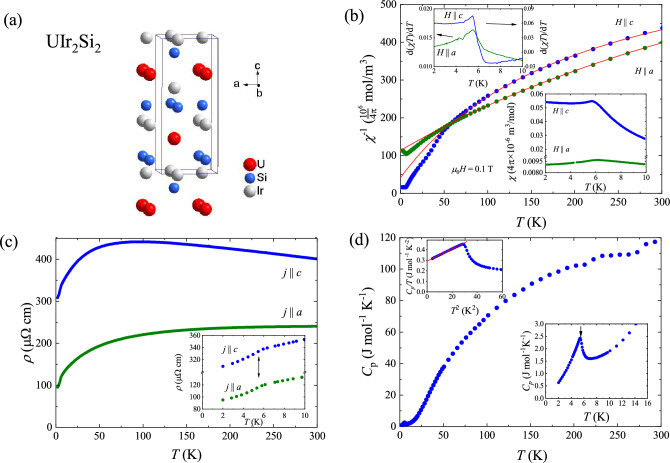
(**a**) Crystal structure of $\hbox {UIr}_{2} \hbox {Si}_{2}$. (**b**) Temperature dependences of the reciprocal molar magnetic susceptibility of single crystalline $\hbox {UIr}_{2} \hbox {Si}_{2}$ recorded in a magnetic field 0.1 T applied along *a* and *c* axes of the tetragonal unit cell. Solid line represents fit of the modified Curie-Weiss law. The lower inset shows the low-temperature magnetic susceptibility data. The upper inset presents derivative curves d($\chi T)$/d*T*. (**c**) Temperature variation of the electrical resistivity of single-crystalline $\hbox {UIr}_{2} \hbox {Si}_{2}$ measured with the electric current flowing along two main crystallgraphic directions. The inset presents low-temperature data. The arrows mark the Néel temperature. (**d**) Specific heat of $\hbox {UIr}_{2} \hbox {Si}_{2}$ as a function of the temperature. The lower inset shows the low-temperature region. The arrow marks the magnetic phase transition temperature. The upper inset presents the data plotted as $C/T(T^{2})$. The solid line is a linear fit described in the text.

X-ray diffraction measurements performed on a single crystal of $\hbox {UIr}_2 \hbox {Si}_2$ confirmed that the compound crystallizes with the tetragonal primitive $\hbox {CaBe}_{2} \hbox {Ge}_{2}$-type unit cell (space group *P*4/*nmm*). Although the crystal structure is centrosymmetric, the U atoms are located in a non-centrosymmetric position $\hbox {C}_{4v}$ (4*mm* in Hermann-Mauguin notation). The crystal structure of $\hbox {UIr}_{2} \hbox {Si}_{2}$ is shown in Fig. 1a. To refine the crystal structure, a two-step approach was employed. First, the initial assumption that all crystallographic positions were fully occupied was made. Then, a slight crystallographic disorder was introduced, the presence of which is reflected in other physical properties, as discussed below. It should be recalled that the previous reports revealed crystallographic disorder in $\hbox {UIr}_{2} \hbox {Si}_{2}$ due to understoichiometry of Ir^18^.

Four models of the crystal structure of $\hbox {UIr}_{2} \hbox {Si}_{2}$ were considered: (i) deficiency of both Ir and Si atoms at the 2*c* positions, (ii) deficiency of Ir and Si at the 2*c* position with some admixture of Si at the Ir 2*c* site and not fully occupied Si position, (iii) slight mixing of Ir and Si at the 2*c* atomic positions with the assumption that composition of the crystal is a nominal one, (iv) small admixture of Si at the Ir 2*c* position. All these models are reasonable and give very similar refinement parameters. However, owing to the EDX reasults, that show an excess of Si, the model (iv), which gives the largest excess of Si, was considered as the most probable. The details of the refinement and the obtained crystallographic data are given in Tables 1, 2, and 3.

**Table 1:** Crystallographic and structure refinement data for $\hbox {UIr}_{2} \hbox {Si}_{2}$.

Compound	$\hbox {UIr}_{2} \hbox {Si}_{2}$
Space group	*P*4/*nmm*
Unit cell dimensions	*a*=4.0834(2) Å
	*b*=4.0834(2) Å
	*c*=9.8247(7) Å
Volume	163.82(2) Å$^{3}$
Formula weight	676.97 g/mol
Calculated density	13.724 g/$\hbox {cm}^{3}$
Absorption coefficient	130.378 $\hbox {mm}^{-1}$
$\theta$ range for data collection	4.148$^{\circ }$ - 28.308$^{\circ }$
Ranges in *hkl*	$-5\leqslant h \leqslant 5$
	$$-5\leqslant k \leqslant 4$$
	$$-12 \leqslant l \leqslant 12$$
Reflections collected/unique	1311/151
Completeness to $\theta$ = 25.242$^{\circ }$	99.1%
Refinement method	Full-matrix least-squares on F${^2}$
Refined parameters	16
Goodness of fit on $\hbox {F}^{2}$	1.151
Final R indices [$I \ge 2 \sigma (I)$]	R${_1}$=0.0205, wR${_2}$=0.0398
R indices (all data)	R${_1}$=0.0249, wR${_2}$=0.0408
Extinction coefficient	0.0044(4)
Largest diff. peak and hole	1.618 and −1.743 e/Å$^{-3}$

**Table 2:** Atomic coordinates, atomic populations, and equivalent isotropic thermal displacement parameters for $\hbox {UIr}_{2} \hbox {Si}_{2}$. $U _{eq}$ is defined as one third of the trace of the orthogonalized $U _{ij}$ tensor.

Atom	Site	*x*	*y*	*z*	Occ.	$$U_{eq}$$ $$(10^{-3}$$Å$$^{2})$$
U1	2*c*	0.25	0.25	0.74727(9)	1 (fixed)	11.0(3)
Ir1	2*a*	0.75	0.25	0	1 (fixed)	10.0(3)
Ir2	2*c*	0.25	0.25	0.37234(10)	0.988(5)	10.7(4)
Si2A	2*c*	0.25	0.25	0.37234(10)	0.012(5)	10.7(4)
Si1	2*b*	0.75	0.25	0.5	1 (fixed)	10.7(14)
Si2	2*c*	0.25	0.25	0.1329(7)	1 (fixed)	11.2(14)

**Table 3:** Anisotropic thermal displacement parameters for the atoms in $\hbox {UIr}_{2} \hbox {Si}_{2}$ (in 10$^{-3}$Å$^{2}$). The anisotropic displacemant factor exponent takes the form: $-2 \pi ^{2} \left[ h^{2}a^{* 2} U_{11}+\cdots +2 hka ^{*} b ^{*} U_{12} \right]$.

	$U _{11}$	$U _{22}$	$U _{33}$	$U _{12}$	$U _{23}$	$U _{13}$
U1	10.7(4)	10.7(4)	11.6(5)	0	0	0
Ir1	9.3(4)	9.3(4)	11.3(5)	0	0	0
Ir2	10.8(4)	10.8(4)	10.7(5)	0	0	0
Si2A	10.8(4)	10.8(4)	10.7(5)	0	0	0
Si1	9(2)	9(2)	13(3)	0	0	0
Si2	11(2)	11(2)	12(3)	0	0	0

### Low-field properties

Figure 1b shows the temperature dependence of the inverse molar magnetic susceptibility, $\chi ^{-1} (T)$ of $\hbox {UIr}_{2} \hbox {Si}_{2}$ measured in a magnetic field of 0.1 T applied parallel and perpendicular to the *c* axis of the tetragonal unit cell. It is noteworthy that the variation measured along the *c* axis is strongly bent at lower temperatures and, remarkably, two $\chi (T)$ curves cross each other at about 60 K. Such a behavior, marking changes in magnetocrystalline anisotropy, has been reported previously for this compound^14^. Interestingly, it was also observed for $\hbox {UIrSi}_{3}$, which crystallizes with a closely related crystal structure^23^. Above 50 K, both variations follow a modified Curie-Weiss (MCW) law: $\chi (T) = \chi _0 + \dfrac{\mu _{eff}^{2}}{8(T-\theta _P)}$, where $\mu _{eff}$ is the magnetic effective moment, $\theta _{P}$ stands for the paramagnetic Curie temperature, and $\chi _{0}$ is the temperature independent Pauli-like susceptibility. The least-square fitting of the formula to the experimental data yielded $\chi _0 = 9\times 10 ^{-4}$ emu/mol, $\mu _{eff} = 2.17 \ \mu _{B}$, and $\theta _{P} = -75 \ {\rm K}$ for $H \parallel a$, and $\chi _0 = 1.4\times 10 ^{-3}$ emu/mol, $\mu _{eff} = 1.5 \ \mu _{B}$, and $\theta _{P} = -12 \ {\rm K}$ for $H \parallel c$. The $\mu _{eff}$ is much smaller than those predicted for free $\hbox {U}^{3+}$ and $\hbox {U}^{4+}$ ions (3.62 and 3.58 $\mu _{B}$, respectively), which can be ascribed to crystal field effects and possibly also partial delocalization of U 5*f* electrons. $\theta _P$ is negative and has a large absolute value similar to those reported previously^19^.

The low-temperature dependencies of the magnetic susceptibility of single-crystalline $\hbox {UIr}_{2} \hbox {Si}_{2}$, are shown in the lower inset of Fig. 1b. The compound exhibits a distinct magnetocrystalline anisotropy with the magnetization component measured along the *c* axis being distinctly larger than that taken along the *a* axis. This result is in agreement with the previous reports^14,18^ and the neutron diffraction experiments^19^. At low temperatures, both $\chi (T)$ variations show maxima signaling the onset of the antiferromagnetic ordering. Below the Néel temperature, $\chi _{c}$ does not decrease monotonically, but forms a shallow minimum followed by a slight upturn. Such a feature is not expected for simple antiferromagnets and may indicate more complicated magnetic structure in $\hbox {UIr}_{2} \hbox {Si}_{2}$ than reported before^19^. Very similar behavior of the magnetic susceptibility was observed also by Vernière^18^. The Néel temperature determined as the inflection points on the $\chi T (T)$ variations^24^ equals 5.5 K (see the upper inset to Fig. 1b), i.e within the reported previously range (4.9–6 K)^14,18^.

The temperature dependencies of the electrical resistivity of single-crystalline $\hbox {UIr}_{2} \hbox {Si}_{2}$, $\rho _{\parallel } (T)$ and $\rho _{\perp } (T)$, measured with the electric current flowing along and perpendicular to the *c* axis, respectively, are shown in Fig. 1c. The resistivity shows a distinct anisotropy with the component measured along the four-fold axis being much larger than that measured perpendicular to it. This feature is characteristic of $\hbox {UT}_{2} \hbox {M}_{2}$ compounds that adapt the $\hbox {CaBe}_{2} \hbox {Ge}_{2}$-type structure^25^. Closely related intermetallics crystallizing with the $\hbox {ThCr}_{2} \hbox {Si}_{2}$-type unit cell show a different anisotropy with smaller resistivity along the tetragonal axis^25^. At room temperature, $\rho _{\parallel } = 400 \ \mu \Omega \ {\rm cm}$ and $\rho _{\perp } = 240 \ \mu \Omega \ {\rm cm}$. With decreasing temperature $\rho _{\parallel }$ initially increases, undergoes a broad maximum at 80 K and then decreases down to 2 K. In turn, $\rho _{\perp }$ decreases monotonically down to the lowest temperatures. A significant feature of both $\rho (T)$ components is a rather high magnitude of the residual resistivity, being $\rho _{\parallel }=309\ \mu \Omega \ {\rm cm}$ and $\rho _{\perp }=95\ \mu \Omega \ {\rm cm}$ at 2 K. The residual resistivity ratio RRR defined as $\rho (300 \ {\rm K})/\rho (2 \ {\mathrm K})$ equals to 1.3 and 2.5 for $j \parallel c$ and $j \perp c$, respectively. Large values of $\rho (2 \ {\rm K})$ and thus small values of RRR can be linked to the atomic disorder at the Ir/Si positions in the studied crystal. As shown in the inset to Fig. 1c, at low temperatures both resistivity components form a knee-like anomaly associated with the magnetic ordering. The Néel temperature defined as the inflection points of these curves is equal to 5.3 K for both directions of the electric current. This value is very close to that obtained from the magnetic susceptibility data.

Figure 1d presents the temperature dependence of the specific heat of $\hbox {UIr}_{2} \hbox {Si}_{2}$, $C_p(T)$. At room temperature, $C_p$ is nearly saturated at a value of 120 J $\hbox {mol}^{-1} \hbox {K}^{-1}$, which is close to the theoretical Dulong-Petit limit 3nR = 125 J $\hbox {mol}^{-1} \hbox {K}^{-1}$ (where n = 5 is the number of atoms per molecule and R represents the universal gas constant). The onset of the antiferromagnetic state manifests itself as a distinct $\lambda$-like anomaly (see the lower inset to the figure). As can be inferred from the upper inset to Fig. 1d, the specific heat data can be well described below $T_{N}$ by the formula $C_{p} = \gamma T + \beta T^{3}$, where the first term represents the electronic contribution and the second one is the sum of the contributions due to antiferromagnetic magnons and phonons. The least-square fitting of the above equation to the experimental data yielded the parameters $\gamma = 292 \ {\rm J \, mol}^{-1}{\rm K}^{-2}$ and $\beta = 5.8 \times 10^{-3}\ {\rm J \, mol}^{-1} \, {\rm K}^{-4}$. The value of $\gamma$ is very close to that reported in the literature^14,19^. Its enhanced magnitude can be attributed to strong electronic correlations but may also result from the atomic disorder in the crystal structure of $\hbox {UIr}_{2} \hbox {Si}_{2}$ revealed in the X-ray diffraction experiment and supported by the electrical resistivity data.

**Figure 2:**
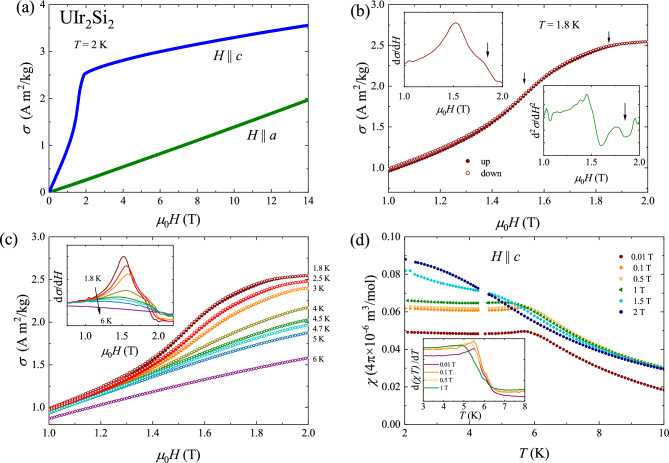
(**a**) Magnetization of $\hbox {UIr}_{2} \hbox {Si}_{3}$ measured as a function of the external magnetic field oriented along *a* and *c* axes of the tetragonal unit cell, at a constant temperature of 2 K. (**b**) The magnetization measured with increasing (full circles) and decreasing (open circles) magnetic field applied along the *c* axis, close to the magnetic transitions. Arrows mark $H_{c1}$ and $H_{c2}$. Insets present first and second order field derivatives of the magnetization taken with increasing field. Arrows mark $H_{c2}$. (**c**) Field dependencies of the magnetization measured along the *c* axis with increasing and decreasing magnetic field (full and open symbols respectively) at several temperatures. The inset presents magnetic field derivatives of the magnetization. (**d**) Temperature dependencies the magnetic susceptibility measured at various magnetic fields oriented along the *c* axis. The inset presents the temperature derivatives ($d\chi )T/dT$.

### Metamagnetic transition

Figure 2a presents the magnetic field dependencies of the magnetization $\sigma (H)$ of the $\hbox {UIr}_{2} \hbox {Si}_{2}$ single crystal measured at constant temperature of 2 K in a magnetic field applied along two principal directions. The variation taken along the *a* axis, $\sigma _a (H)$, being the hard magnetic direction, is straight-linear up to a limiting field of 14 T. In contrast, the magnetization measured with the magnetic field applied along the *c* axis, $\sigma _c (T)$, shows a rapid increase near 1.5 T, indicating a metamagnetic phase transition. Remarkably, $\sigma _c (T)$ does not tend to saturate in strong magnetic fields, and at 14 T attains a value that corresponds to the magnetic moment of $0.43 \ \mu _B$. This is much larger than the magnetic moment of 0.1 $\mu _B$/U atom revealed for $\hbox {UIr}_{2} \hbox {Si}_{2}$ by neutron diffraction^19^. It has been suspected that magnetic field splits the excited crystal field doublet, located close to the ground state, and such a mixing with the excited crystal field level leads to enhanced magnetism^20^. Extrapolation of the $\sigma _a (H)$ and $\sigma _c (H)$ variations beyond investigated field range results in crossing near 80 T, that can be considered as a rough estimation of the magnetocrystalline anisotropy field. This value is similar to the magnetocrystalline anisotropy field of 59 T, derived for a closely related compound $\hbox {UIrSi}_{3}$^23^.

Figure 2b shows the magnetization data taken at 1.8 K in magnetic field in the range 1–2 T applied parallel to the four-fold axis. The left and right insets to the figure show the first and second order field derivatives of the magnetization $d \sigma /d T$ and $d \sigma ^{2} /d^{2}T$, respectively, taken with increasing magnetic field. As can be seen, the $\sigma (H)$ variations do not overlap and show a very narrow hysteresis marking the first order of the transition, as expected for metamagnets^26^. The critical field $H_{c1}$ was defined as an inflection point on the $\sigma (H)$ variation (note a maximum in the first order derivative $d\sigma /d H$) and amounts to 1.52 T. At the $d\sigma /d H$ curve, above the maximum, there can be observed a distinct bump that can be linked to a slight kink in $\sigma (H)$ occuring near 1.85 T (note a minimum in the second-order derivative). Such a feature suggests a spin-flop character of the metamagnetic transition, in agreement with the relatively small magnetic anisotropy in $\hbox {UIr}_{2} \hbox {Si}_{2}$. In stronger fields another phase transition, to the field-induced paramagnetic phase, can be expected, where the alignment of the magnetic moments changes continuously towards the easy direction^26,27^.

**Figure 3:**
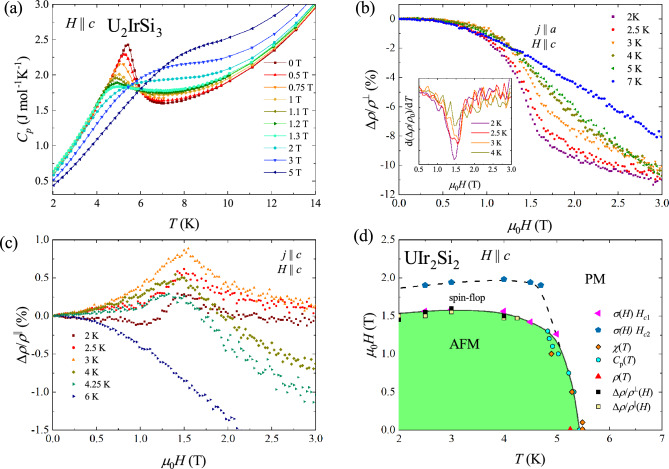
(**a**) Specific heat of $\hbox {UIr}_{2} \hbox {Si}_{2}$ measured at low temperatures with magnetic field applied along the *c* axis. (**b**) Magnetic field dependence of the transverse magnetoresistivity measures at different temperatures with $j\parallel a$ and $H \parallel c$. (**c**) Longitudinal magnetoresistivity as a function of magnetic field measured at several temperatures with $j\parallel H \parallel c$. (**d**) Magnetic phase diagram of $\hbox {UIr}_{2} \hbox {Si}_{2}$.

Figure 2c presents the magnetization isotherms measured at different temperatures. The inset shows the field derivatives of the magnetization. For each isotherm, the critical field $H_{c1}$ was defined as a maximum in $d\sigma /dH$, while $\hbox {H}_{c2}$ was defined as a kink on the $\sigma (H)$ variation (an inflection point in $d\sigma /dH$ above the hump). It was found that $H_{c1}$ hardly depends on temperature up to 4 K, and then slightly decreases. In stronger fields, $H_{c2}$ does not change up to 4.7 K, and cannot be identified at higher temperatures. The $\sigma (H)$ variation measured at 6 K does not show any anomalies, and is typical of paramagnets. The so-determined values of the critical fields are plotted on a magnetic phase diagram presented in Fig. 3d.

Figure 2d shows the temperature dependencies of the magnetic susceptibility $\chi (T)$ measured in different magnetic fields applied along the *c* axis. With increasing field up to 1 T, the maximum associated with the magnetic ordering shifts slightly towards lower temperatures, as expected for antiferromagnets. At stronger fields, above the metamagnetic transition field, the susceptibility increases down to the lowest temperatures measured, and does not show any anomaly. The Néel temperature, determined as a maximum on the d$(\chi T)$/d*T* variation (see the inset to Fig. 2c), is equal to 5.5 K for a magnetic field of 0.01 T. It hardly changes up to 0.5 T, and decreases down to 5 K for 1 T. The so-obtained data were added to Fig. 3d.

The low-temperature dependencies of the specific heat of $\hbox {UIr}_{2} \hbox {Si}_{2}$ taken in zero and finite magnetic fields applied along the *c* axis are presented in Fig. 3a. With increasing field the $\lambda$-like maximum associated with the magnetic ordering shifts towards lower temperatures, broadens, and becomes smaller, in a manner typical of antiferromagnets. In a field of 1.5, that is the critical field of the metamagnetic transition, the maximum is superimposed on a broad hump. In stronger magnetic fields, this hump shifts towards higher temperatures, as expected for field-polarized paramagnets. The Néel temperature, defined as the maximum in the $C_p(T)$ variation, gradually shifts towards lower temperatures with increasing the magnetic field strength, and at 1.3 T $T_N$ is equal to 4.92 K. These data have also been included in the phase diagram shown in Fig. 3d.

The magnetic phase transitions in $\hbox {UIr}_{2} \hbox {Si}_{2}$ are also well seen in the electrical transport data. Figure 3b, c present the field dependencies of the magnetoresistivity of the studied crystal, defined as $\dfrac{\Delta \rho }{\rho _0} = \dfrac{\rho (H)-\rho (0)}{\rho (0)}$, measured with the magnetic field applied along the *c* axis and electric current *j* flowing parallel and perpendicular to the c axis, respectively. As can be seen in Fig. 3b, with increasing magnetic field, the transverse magnetoresistivity $\Delta \rho /\rho _0^{\perp }$ measured below ${T_N}$ initially slightly decreases. The spin-flop transition manifests itself as a sudden drop in $\Delta \rho / \rho _0 ^{\perp }$, down to a value of $-11$% at 2 K in a field of 3 T. The critical field $H_{c1}$ can be determined for each isotherm by inspecting the field derivatives of the magnetoresistivity (cf. the inset to Fig. 3b). Generally, $H_{c1}$ does not change much up to 4 K. It should be noted that no clear signature of the upper critical field $H_{c2}$ is seen in the resistivity data. In the paramagnetic state, the transverse magnetoresistivity decreases monotonically over the entire field range and does not show any feature, as expected for paramagnets.

As apparent from Fig. 3c, the longitudinal magnetoresistivity $\Delta \rho /\rho _0 ^{\parallel }(H)$ is much smaller than the transverse one. The metamagnetic phase transition manifests itself as a maximum in $\Delta \rho /\rho _0 ^{\parallel } (H)$. The variation measured at 2 K shows a distinct feature of 0.25 % at $\mu _0 H_{c1} = 1.5$ T. Interestingly, at 3 K, the magnitude of the maximum associated with the metamagnetic transition increases up to 0.9%. Further increase of temperature results in decrease of the maximum. The feature marking $H_{c1}$ remains almost temperature independent up to 4.25 K. In the paramagnetic region, the longitudinal magnetoresistivity does not show any anomaly, alike $\Delta \rho / \rho _{0}^{\perp } (H)$. The so-obtained data points were added to Fig. 3d.

## Conclusions

The silicide $\hbox {UIr}_{2} \hbox {Si}_{2}$ crystallizes with a locally non-centrosymmetric tetragonal crystal structure of the $\hbox {CaBe}_{2} \hbox {Ge}_{2}$ type. The compound orders antiferromagnetically below the Néel temperature of 5.5 K. The antiferromagnetic phase transition manifests itself as a maximum in the magnetic susceptibility, a knee-like anomaly in the electrical resistivity and a distinct $\lambda$-like anomaly in the specific heat. $\hbox {UIr}_{2} \hbox {Si}_{2}$ shows relatively small magnetic anisotropy with the easy magnetic direction oriented along the *c* axis. The magnetic properties are fairly robust in magnetic fields up to 14 T applied along the *a* axis of the tetragonal unit cell. On the contrary, the compound undergoes a metamagnetic transition in fields applied along the *c* axis. At 1.8 K, the critical field is about 1.5 T. This transition gives rise to distinct features in the low-temperature field dependencies of the magnetization and the magnetoresistance. Furthermore, in magnetic fields stronger than 1.5 T, the temperature variations of the magnetization and the heat capacity distinctly change their character.

Combining the data from the magnetization, specific heat, electrical resistivity, and magnetoresistivity measurements, the magnetic phase diagram of $\hbox {UIr}_{2} \hbox {Si}_{2}$ was constructed, which is presented in Fig. 3d. In zero field, the investigated crystal was found to order antiferromagnetically at $T_N$ = 5.5 K. Below the Néel temperature, the field-induced metamagnetic transition to the spin-flop state was established, followed by another transition to the field-induced paramagnetic phase. At 1.8 K, the critical fields are relatively small being $\mu _0 H_{c1}$ = 1.52 T and $\mu _0 H_{c2}$ = 1.85 T at 1.8 K.

The magnetic phase diagram derived for $\hbox {UIr}_{2} \hbox {Si}_{2}$ can be compared with that obtained for a closely related compound $\hbox {UIrSi}_{3}$. The latter crystallizes with a similar crystal structure, also being derivative of the $\hbox {BaAl}_{4}$-type, in which the U atoms are located at a non-centrosymmetric position^23,28^. The magnetic anisotropy in the latter compound is similar, with the easy magnetic direction along the *c* axis. Also the magnetocrystalline anisotropy field estimated for both compounds has similar values. $\hbox {UIrSi}_{3}$ orders antiferromagnetically at $T_N = 41.7 \, {\mathrm K}$, which is much larger than $T_N$ of $\hbox {UIr}_{2} \hbox {Si}_{2}$. At low temperatures, $\hbox {UIrSi}_{3}$ undergoes a first-order metamagnetic transition, characterized by rather small critical field, compared to its $T_{N}$ (7.3 T at 2 K). At the critical field, the measured physical characteristics show wide hysteresis loops (different from $\hbox {UIr}_{2} \hbox {Si}_{2}$), which narrow and shift towards lower fields with increasing magnetic field strength. With increasing temperature, the character of the transition changes to the second order, and a tricritical point in the phase diagram occurs at $T=$ 28 K and $\mu _{0}H=$ 5.8 T.

## Methods

A single crystal of $\hbox {UIr}_{2} \hbox {Si}_{2}$ was grown by the Czochralski pulling technique in an ultra-pure Ar atmosphere using a tetra-arc furnace (GES Corporation). The starting components were high-purity elements (U-3N,Ir-3N, and Si-6N). The obtained crystal was a rod of approximately 4 mm in diameter and 15 mm in length.

The crystal homogeneity was checked by energy dispersive X-ray spectroscopy analysis performed on an FEI scanning electron microscope equipped with an EDAX Genesis XM4 spectrometer.

The Oxford X’Calibur four-circle single-crystal diffractometer, equipped with a CCD camera and graphite-monochromated MoK$\alpha$ radiation ($\lambda$ = 0.71073 Å), was used to collect X-ray diffraction data at room temperature from a single crystal with dimensions of 0.08 $\times$ 0.07 $\times$ 0.05 mm. The data collection and reduction were performed with CrysAlis PRO software (1.171.39.46 and 1.171.41.93a, Rigaku Oxford Diffraction, 2018 and 2020). Absorption was corrected based on Gaussian integration over a multifaceted crystal model. The structure was solved by direct methods and refined using the SHELX-2014 crystallographic software package^29^ in cooperation with the graphical user interface of Olex2^30^.

Magnetic properties measurements were performed in the temperature range 1.8–300 K in magnetic fields up to 7 T using a Quantum Design MPMS-7 superconducting quantum interference device (SQUID) magnetometer, and up to 14 T employing a Quantum Design PPMS-14 platform equipped with a vibrating sample magnetometer. The heat capacity was measured in the temperature interval 2-300 K and in magnetic fields up to 5 T, using a relaxation technique. The temperature and magnetic field variations of the electrical resistivity were studied from 2 to 300 K, in magnetic fields up to 14 T, employing a standard ac four-probe method. For these measurements a Quantum Design PPMS-9 and PPMS-14 platforms were employed.

## Data Availability

The datasets used and/or analyzed during the current study available from the corresponding author on reasonable request.

## References

[1] Szytuła A (1992). Magnetic properties of 1:2:2 rare-earth and actinide intermetallics. J. Alloys Compd..

[2] Süllow S (2000). Disorder to order transition in the magnetic and electronic properties of URh$$_{2}$$Ge$$_{2}$$. Phys. Rev. B.

[3] Süllow S (2008). Electronic localization and two-dimensional metallic state in UPt$$_{2}$$Si$$_{2}$$. J. Phys. Soc. Jpn..

[4] Mydosh JA, Oppeneer PM, Riseborough PS (2020). Hidden order and beyond: An experimental-theoretical overview of the multifaceted behavior of URu$$_{2}$$Si$$_{2}$$. J. Phys. Condens. Matter.

[5] Palstra T, Menovsky A, Nieuwenhuys G, Mydosh J (1986). Magnetic properties of the ternary compounds CeT$$_{2}$$Si$$_{2}$$ and UT$$_{2}$$Si$$_{2}$$. J. Magn. Magn. Mater..

[6] Dalichaouch Y, Maple MB, Torikachvili MS, Giorgi AL (1989). Ferromagnetic instability in the heavy-electron compound URu$$_{2}$$Si$$_{2}$$ doped with Re or Tc. Phys. Rev. B.

[7] Matsuda TD (2003). Crystal and magnetic structure in the itinerant 5f antiferromagnet UCr$$_{2}$$Si$$_{2}$$. J. Phys. Condens. Matter.

[8] Matsuda TD (2003). Single crystal growth and structural and magnetic properties of the uranium ternary intermetallic compound UCr2Si2. J. Phys. Soc. Jpn..

[9] Szytuła A, Siek S, Leciejewicz J, Zygmunt A, Ban Z (1988). Neutron diffraction study of UT$$_{2}$$X$$_{2}$$ (T = Mn, Fe, X = Si, Ge) intermetallic systems. J. Phys. Chem. Solids.

[10] Chełmicki L, Leciejewicz J, Zygmunt A (1985). Magnetic properties of Ut2Si2 and Ut2Ge2 (t = Co, Ni, Cu) intermetallic systems. J. Phys. Chem. Solids.

[11] Svoboda P (2002). Magnetic phase diagram and critical scattering of UNi$$_{2}$$Si$$_{2}$$. Phys. B Condens. Matter.

[12] Matsuda TD (2005). Electrical and magnetic properties of a single crystal UCu2Si2. J. Phys. Soc. Jpn..

[13] Troć R (2013). Phenomenological crystal-field model of the magnetic and thermal properties of the Kondo-like system UCu$$_{2}$$Si$$_{2}$$. Phys. Rev. B.

[14] Dirkmaat AJ (1990). Magnetic, thermal, and transport properties of UIr$$_{2}$$Si$$_{2}$$. Phys. Rev. B.

[15] Steeman RA (1990). Hybridisation effects in UPt$$_{2}$$Si$$_{2}$$. J. Phys. Condens. Matter.

[16] Tabata C (2016). Peculiar magnetism of UAu$$_{2}$$Si$$_{2}$$. Phys. Rev. B.

[17] Amorese A (2020). From antiferromagnetic and hidden order to Pauli paramagnetism in UM$$_{2}$$Si$$_{2}$$ compounds with 5f electron duality. Proc. Natl. Acad. Sci..

[18] Vernière A (1995). Low temperature structural and physical behaviour of UIr$$_{2}$$Si$$_{2}$$ single crystal. Phys. B Condens. Matter.

[19] Vernière A (1996). Magnetic structure and physical properties of the heavy fermion UIr$$_{2}$$Si$$_{2}$$. J. Magn. Magn. Mater..

[20] Vernière A, Boucherle J-X, Lejay P, Gillon B (1999). Field-induced magnetic form factor in UIr2Si2. Physica B Condens. Matter.

[21] Ikeda S (2006). Effect of pressure on the electronic state in antiferromagnets Upt$$_{2}$$Si$$_{2}$$ and UIr$$_{2}$$Si$$_{2}$$. J. Phys. Soc. Jpn..

[22] Matsuda M (2021). Elastic properties and crystalline-electric-field effects of UIr$$_{2}$$Si$$_{2}$$. JPS Conf. Proc..

[23] Valenta J (2018). Antiferromagnetism and phase transitions in noncentrosymmetric UIrS3. Phys. Rev. B.

[24] Fisher ME (1962). Relation between the specific heat and susceptibility of an antiferromagnet. Philos. Mag. J. Theor. Exp. Appl. Phys..

[25] Steeman R (1990). Anisotropic resistivity of (Ce, U)T$$_{2}$$X$$_{2}$$ compounds in relation to their crystal structure. Phys. B Condens. Matter.

[26] Stryjewski E, Giordano N (1977). Metamagnetism. Adv. Phys..

[27] Buschow KHJ, de Boer FR (2004). Physics of Magnetism and Magnetic Materials.

[28] Honda F (2019). Magnetotransport as a probe of phase transformations in metallic antiferromagnets: The case of UIrSi3. Phys. Rev. B.

[29] Sheldrick GM (2015). Crystal structure refinement with SHELXL. Acta Crystallogr. A.

[30] Dolomanov O, Bourhis L, Gildea R, Howard J, Puschmann H (2009). OLEX2: A complete structure solution, refinement and analysis program. J. Appl. Cryst..

